# Chemokine‐like factor 1 (CKLF1) aggravates neointimal hyperplasia through activating the NF‐κB /VCAM‐1 pathway

**DOI:** 10.1002/2211-5463.12942

**Published:** 2020-08-14

**Authors:** Xinnong Liu, Chengjia Qu, Yongbao Zhang, Jie Fang, Lequn Teng, Rujiao Zhang, Xiangyu Zhang, Chenyang Shen

**Affiliations:** ^1^ Vascular Surgery Center State Key Laboratory of Cardiovascular Disease National Center for Cardiovascular Diseases Fuwai Hospital Chinese Academy of Medical Sciences and Peking Union Medical College Beijing China; ^2^ College of Clinical Medicine Hebei University Baoding China; ^3^ Department of General Surgery Sir Run Run Shaw Hospital Zhejiang University School of Medicine Hangzhou China

**Keywords:** chemokine‐like factor 1, neointimal hyperplasia, NF‐κB, restenosis, VCAM‐1

## Abstract

Neointimal hyperplasia (NIH) is a complicated inflammatory process contributing to vascular restenosis. The present study aimed to explore whether chemokine‐like factor 1 (CKLF1) aggravates NIH via the nuclear factor‐kappa B (NF‐κB)/vascular cell adhesion molecule‐1 (VCAM‐1) pathway. We found the expression of CKLF1 and VCAM‐1 significantly increased in human carotid plaques compared to the control. *In vivo*, CKLF1 overexpression induced a thicker neointimal formation and VCAM‐1 expression was correspondingly upregulated. *In vitro*, CKLF1 activated NF‐κB and induced VCAM‐1 upregulation in human aortic smooth muscle cells (HASMCs). Functional experiments demonstrated that CKLF1 promoted monocyte adhesion and HASMC migration via VCAM‐1. These results suggest CKLF1 accelerates NIH by promoting monocyte adhesion and HASMC migration via the NF‐κB/VCAM‐1 pathway. Our findings contribute to a better understanding of the mechanisms underlying the causality of CKLF1 on NIH and could prove beneficial in designing therapeutic modalities with a focus on CKLF1.

## Graphical abstract

Neointimal hyperplasia (NIH) plays a pivotal role in vascular restenosis after revascularization. We previously identified that chemokine‐like factor 1 (CKLF1) could aggravate NIH by arresting smooth muscle cells in G2/M phase and preventing apoptosis via phosphoinositide 3‐kinase/AKT/nuclear factor‐kappa B signaling. Here, we demonstrate that CKLF1 promotes monocyte adhesion and smooth muscle cell migration via VCAM‐1. Our work furthers our understanding of how CKLF1 contributes to NIH causality.

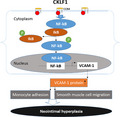

**Abbreviations**
α‐SMA: α‐smooth muscle actin
CKLF1: chemokine‐like factor 1
HASMC: human aortic smooth muscle cell
HE: hemotoxylin and eosin
NaCl/Pi: phosphate‐buffered saline
NF‐κB: nuclear factor‐kappa B
NIH: neointimal hyperplasia
PDTC: pyrrolidinedithiocarbamic acid
PI3K: phosphoinositide 3‐kinase
SMC: smooth muscle cell
VCAM‐1: vascular cell adhesion molecule‐1

Neointimal hyperplasia (NIH) plays a pivotal role in vascular restenosis after revascularization, leading to repeated interventions and a substantial economic burden [1]. The restenosis rate can be up to 30–50% for patients treated with balloon angioplasty or bare‐metal stents, and still up to 12–20% for patients who received drug‐eluting stents [2, 3]. Although the mechanisms are not fully elucidated, current evidence suggests that innate immune‐related inflammatory mechanisms can be involved in the process of NIH [4]. The initial phase of NIH consists of the release of various inflammatory mediators and the recruitment of circulating cells (leukocytes and platelets) to the injured site and subsequently circulating cell activation [5]. In the late phase, NIH is characterized by smooth muscle cell (SMC) proliferation and extracellular matrix formation, which results from the inflammatory mediators released by injured arteries or inflammatory cells [6, 7].

Chemokine‐like factor 1 (CKLF1), as a member of chemokine‐like factor protein family, has detrimental impacts on various biological functions, including chemotactic activities and pulmonary fibrosis [8, 9]. Our previous study has established a novel role of CKLF1 in the progression of human atherosclerosis and NIH after vascular injury [10]. Recently, we identified that CKLF1 could aggravate NIH by arresting SMCs in G2/M phase and preventing apoptosis of SMCs via phosphoinositide 3‐kinase (PI3K)/AKT/nuclear factor‐kappa B (NF‐κB) signaling [11]. Vascular cell adhesion molecule‐1 (VCAM‐1), as a downstream target of NF‐κB signaling, can be expressed by endothelial cells as well as activated SMCs [12, 13]. Several lines of evidence indicate that VCAM‐1 plays a major role in the process of NIH. Experiments in rats showed that VCAM‐1 was required for accumulation of SMCs in neointima formation [14]. Consistent with these data, a clinical study showed that circulating VCAM‐1 was associated with the extent of coronary lesions in patients with atherosclerotic disease [15].

However, the role of NF‐κB/VCAM‐1 signaling in CKLF1‐mediated NIH still remained unclear. Here, we conducted a series of assays to confirm that CKLF1 could accelerate NIH by promoting monocyte adhesion and SMC migration through activating the NF‐κB/VCAM‐1 pathway. This finding may be useful for understanding the detrimental effect of CKLF1 on NIH and beneficial in the development of CKLF1‐target drugs.

## Materials and methods

### Human carotid plaque collection

We collected three carotid plaques from male patients who received carotid endarterectomy. Patients with carotid artery stenosis caused by atherosclerosis were included in the study. The exclusion criteria comprised: refusal to participate in the study, carotid artery stenosis as a result of immunological diseases such as Takayasu arteritis and an active stage of infectious disease. Healthy human radial artery tissues were obtained from donors and served as the control group. Participants provided their written informed consent. The plaques and radial artery tissues collection were conducted in compliance with the Declaration of Helsinki and the study was approved by the Medical Ethical Committee of Fuwai Hospital.

### Animals and treatments

The procedures were performed in accordance with the National Institute of Health Guide for the Care and Use of Laboratory Animals (NIH Publications NO. 85‐23, revised 1996) and the study was approved by the Animal Care and Use Committee of Fuwai hospital. Twenty adult male Sprague–Dawley rats (mean weight 350 g) were purchased from Vital River Laboratory Animal Technology Co., Ltd. (Beijing, China) and housed in a standard laboratory environment. Animals had free access to standard solid claviform food and autoclaved tap water. To simulate an angioplasty in rats, the carotid artery balloon‐injured models were performed with a 2.0‐mm diameter balloon (Medtronic, Dublin, Ireland) as described previously [16]. After balloon injury, rats were randomized into the four groups (five rats per group): Ad‐*CKLF1*, Ad‐*GFP*, Ad‐*shCKLF1* and Ad‐*shRNA scramble*.

Recombinant adenovirus carrying CKLF1 (Ad‐*CKLF1*) or carrying green fluorescence protein (Ad‐*GFP*) was obtained from Shandong ViGenebio Co., Ltd. (Jinan, China). To achieve the highest knockdown efficiency, three pairs of short‐hairpin RNA for CKLF1 were designed and the effective RNA interference knockdown screening was conducted using RT‐PCR as described in our previous study [11]. The optimal sequence was GATCCGCCTCAGTCTGAAATGCTTTGTTCAAGAGACAAAGCATTTCAGACTGAGGCTTTTTTA. The corresponding adenovirus short‐hairpin RNA scramble served as a negative control. The virus (5 × 10^8^ pfu) was injected into injured arteries via the incision of the left external carotid artery and kept *in situ* for 30 min. After 2 weeks, rats were anesthetized by the injection of pentobarbital sodium and 2 cm of the carotid arteries was collected for subsequent experiments.

### Cell culture and adenoviral transfection

Human aortic smooth muscle cells (HASMCs) were purchased from ScienCell (Carlsbad, CA, USA) (catalog no. 6110) and cultured in SMCM medium (catalog no. 1101; ScienCell). Experiments on HASMCs were performed using cells with passage number 3–5. To explore the mechanism through which CKLF1 regulate the expression of VCAM‐1, cells were randomized into four groups (three samples per group): Ad‐*CKLF1*, Ad‐*GFP*, Ad‐*CKLF1* with NF‐κB inhibitor [pyrrolidinedithiocarbamic acid (PDTC), S1808; Beyotime, Shanghai, China] and Ad‐*CKLF1* with dimethylsulfoxide (D2650; Sigma‐Aldrich, St Louis, MO, USA). The cultured HASMCs at 40% confluence were treated with adenovirus particles for 6 h and cultured in complete medium. Ad‐*GFP* was used as the negative control. To explore the effect of VCAM‐1 on the function of HASMCs, cells were randomized into four groups (three samples per group): Ad‐*CKLF1*, Ad‐*GFP*, Ad‐*CKLF1* with VCAM‐1 inhibitor K‐7174 (HY‐12743; MedChem Express, Shanghai, China) and Ad‐*CKLF1* with dimethylsulfoxide.

### Histological and morphometric analyses

Rats were anesthetized with pentobarbital sodium (0.2 mL per 100 g) at 2 weeks after injury. After circulation perfusion with 4% paraformaldehyde, the carotid artery was excised and fixed with 4% paraformaldehyde in phosphate‐buffered saline phosphate buffered saline (NaCl/P_i_) (Gibco, Gaithersburg, MD, USA). The harvested carotid arteries were processed using standard procedures in graded alcohols and xylene and paraffin embedded. Paraffin‐embedded slices were serially sectioned at 4‐μm intervals. The slides were stained using hemotoxylin and eosin (HE) staining. The intimal and medial areas were measured in three sections per rat using imagej, version 1.45 (NIH, Bethesda, MD, USA) and the ratio of intima to media was calculated.

### Immunofluorescence staining

Tissue slides were stained for VCAM‐1 expression using VCAM‐1 antibody (dilution 1:200) purchased from Abcam (Cambridge, MA, USA) (ab134047). At the same time, slides were co‐localized with antibody to α‐smooth muscle actin (α‐SMA) (dilution 1:900, AA132; Beyotime). To detect specific antibody binding, secondary fluorescein‐conjugated antibodies (dilution 1:1000, ab150113 and ab150077; Abcam) were used, and cell nuclei were stained with DAPI (4',6‐diamidino‐2‐phenylindole; C1002; Beyotime). Digital images were recorded using a confocal laser scanning microscope (Leica Microsystems, Wetzlar, Germany). Density values of positive areas were quantified for statistical analysis using imagej, version 1.45.

### Immunohistochemistry

Immunohistochemistry staining was performed in accordance as described in a previous study [17]. Briefly, after blocking in NaCl/P_i_ containing 5% goat serum for 1 h at room temperature, sections were incubated overnight at 4 °C with primary antibody from Abcam (CKLF1: ab180512, dilution 1:100; VCAM‐1: ab134047, dilution 1:500). The next day, signal amplification was performed with horseradish peroxidase‐conjugated goat anti‐rabbit IgG secondary antibody (Zhongshan Jinqiao Biotechnology Co., Ltd., Beijing, China). The stained slides were observed under a microscope, and the density values of positive areas were quantified using integrated optical density values generated by imagej, version 1.45.

### Western blot analyses

Levels of CKLF1, VCAM‐1 and components of NF‐κB pathway were determined using western blotting. Whole cell lysates were prepared using cell lysis buffer for western blot containing protease and phosphatase inhibitor mixture (Roche, Basel, Switzerland). The extracted proteins (5–20 µg per lane) were separated using precast polyacrylamide gel electrophoresis (NuPAGE Bis‐Tris Gels; Thermo Fisher, Walthem, MA, USA) and transferred onto a 0.2‐μm poly(vinylidene difluoride) membrane using the Trans‐Blot® semi‐dry blot system (Bio‐Rad, Hercules, CA, USA). After blocking for 1 h at room temperature with 5% BSA (Beyotime), the membrane was incubated overnight at 4 °C with various primary antibody to CKLF1 (dilution 1:1000, ab180512; Abcam), to VCAM‐1 (NuPAGE Bis‐Tris Gels 1 1000, ab134047; Abcam), to phospho‐p65 (p‐p65, NuPAGE Bis‐Tris Gels 1:1000; CST), to phospho‐IκBa (p‐IκBa, NuPAGE Bis‐Tris Gels 1:1000; Cell Signaling Technology, Beverly, MA, USA) to p65 (dilution 1:1000; Beyotime) and to IκBa (dilution 1:1000; Beyotime). After incubation with its corresponding secondary antibody for 1 h at room temperature, the specific immunoreactive bands were visualized using an ECL detection reagent (RPN2135; GE Healthcare, Chicago, IL, USA) and quantified using molecular imaging software (ProteinTech, Rosemont, IL, USA).

### RNA extraction and quantitative real‐time PCR

Total RNA was extracted from HASMCs transfected with virus particles using TRIzol reagent (Invitrogen, Carlsbad, CA, USA). RNA purity was determined using a ND‐2000 (NanoDrop; Thermo Fisher) by absorbance at 260 and 280 nm (*A*_260/280_). Samples with a *A*_260/280_ ratio higher than 1.8 were used for subsequent detection. Total RNA was reverse‐transcribed into cDNAs using a PrimeScript™ RT reagent kit with gDNA Eraser (RR047; Takara, Shiga, Japan). For human VCAM‐1 amplification, the primers were: forward, 5′‐GGGAAGATGGTCGTGATCCTT‐3′ and reverse, 5′‐TCTGGGGTGGTCTCGATTTTA‐3′; for human GAPDH amplification, the primers were: forward, 5′‐AATGTGTCCGTCGTGGATCTGA‐3′ and reverse, 5′‐GATGCCTGCTTCACCACCTTCTA‐3′. All reactions involved initial denaturation at 95 °C for 20 s, followed by 40 cycles at 95 °C for 1 s and 60 °C for 20 s. Specific mRNA quantification was performed using real‐time PCR using Hieff® qPCR SYBR Green Master Mix (11203ES03; Yeasan, Guangzhou, China) in a real‐time PCR System (QuantStudio 5; Applied Biosystems, Foster City, CA, USA) in accordance with the manufacturer’s instructions. Results with *C*_t_ values lower 30 were included in the subsequent analysis and relative gene expression was determined using the ΔΔCT method [17].

### Monocyte adhesion assays

Monocyte‐HASMC adhesion assays were performed as described previously [18]. U937 cells (monocytes) were purchased from Cell Resource Center of Chinese Academy of Medical Sciences (Beijing, China) and cultured in RPMI‐1640 medium supplemented with 10% fetal bovine serum. After transfection with Ad‐*CKLF1*, HASMCs were incubated with K‐7174 or dimethylsulfoxide for 24 h. U937 cells were prestained with 10 μm DilC_18_(3) (C1036; Beyotime) at 37 °C for 30 min, added to HASMCs culture at a density of 10^6^ cells per well, and incubated for 1 h at 37 °C. The medium was removed, and cells were washed twice with NaCl/P_i_ for removing nonattached cells. HASMC layers with attached monocytes were visualized and measured with a microscope at 200× magnification. The number of attached monocytes was counted in five randomly areas per well from three independent experiments and averaged.

### Cell migration assays

Cell migration assays were performed in a Transwell inserts with a pore size of 8 μm in a 24‐well format (Catalog 3421; Corning Inc., Corning, NY, USA). Once 40% confluence was achieved, HASMCs were transfected with Ad‐*CKLF1*. After transfection, the cell suspension was added into the upper compartment of the chamber (3.0 × 10^5^ cells per insert), whereas the lower compartment was filled with 600 μL of normal culture medium with K‐7174 or dimethylsulfoxide. The cells in the chambers were incubated at 37 °C for 24 h. Non‐migrated cells were removed with cotton swabs. The migrated cells attached on the filter were fixed with 4% paraformaldehyde, stained with crystal violet (C0775; Sigma‐Aldrich) and photographed. Five areas of per culture insert were randomly selected, and the total number of these areas was calculated.

### Statistical analysis

Statistical analyses were performed using prism, version 8 (GraphPad Software Inc., San Diego, CA, USA). The results are expressed as the mean ± SEM. Data were analyzed using Student's *t*‐test when only two groups were compared or using one‐way analysis of variance when more than two groups were compared. *P* < 0.05 was considered statistically significant.

## Results

### CKLF1 and VCAM‐1 are highly expressed in human carotid plaques

**Fig. 1:**
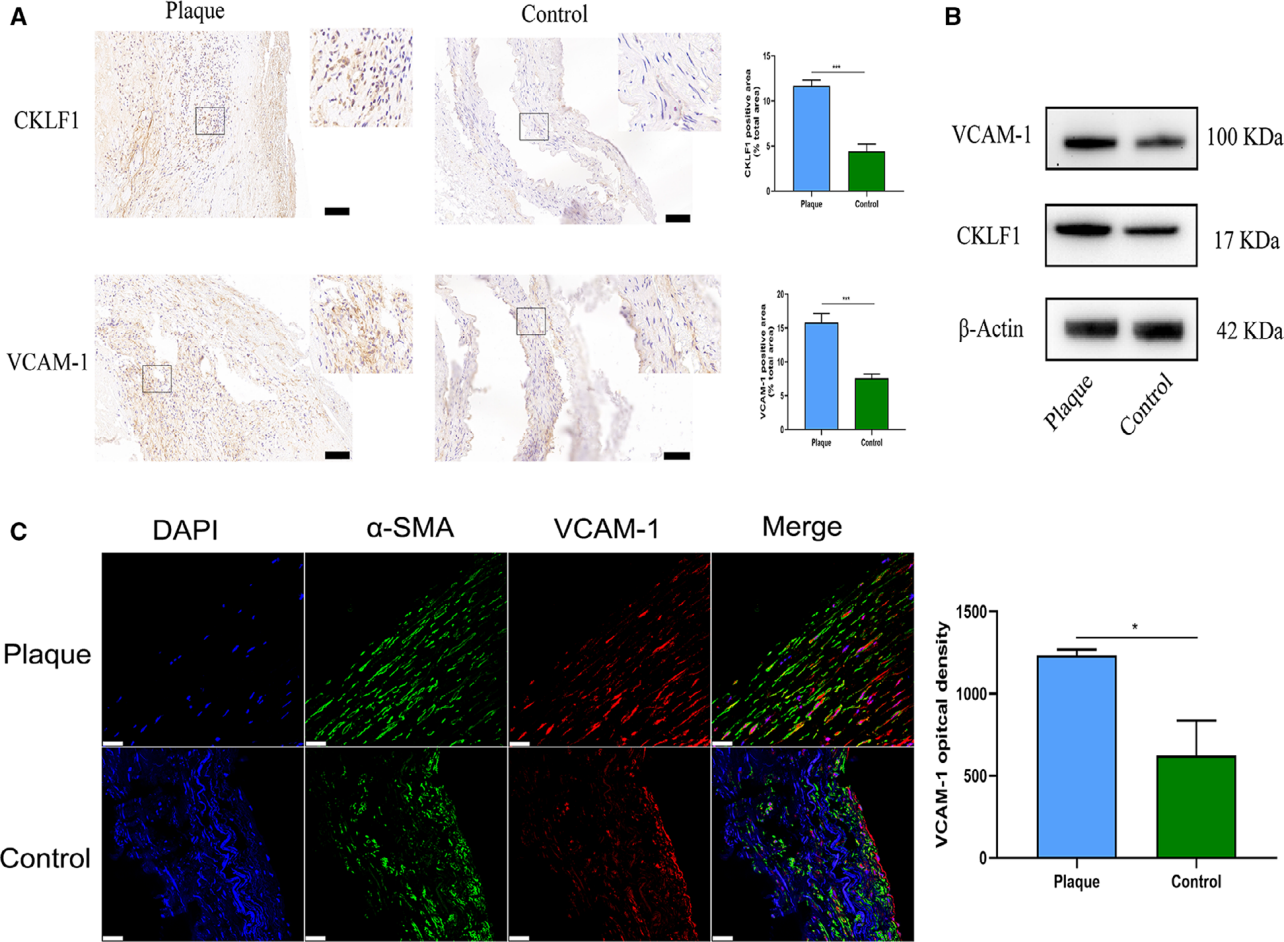
Both CKLF1 and VCAM‐1 are highly expressed in human carotid plaque. (A) Immunohistochemical staining indicated that both CKLF1 and VCAM‐1 expression in human carotid plaque was higher compared to that in the control. Scale bar = 100 μm. Square areas are presented at 40× magnification. (B) Western blotting analysis showed that the levels of CKLF1 and VCAM‐1 in human carotid plaque were obviously increased compared to the control group. (C) Immunofluorescence staining suggested the level of VCAM‐1 was significantly upregulated in human carotid plaque compared to the control group. Smooth muscle cells were co‐localized with specific antibody to α‐SMA and the cell nuclei was stained with DAPI. Scale bar = 25 μm. Density values of positive areas in each slide were used for the statistical analysis. Healthy human radial artery tissue served as the control. Data are shown as the mean ± SEM. Differences between two groups were analyzed by Student's *t*‐test (*n* = 3 independent experiments, *P < 0.05, ***P < 0.001).

The expression of CKLF1 and VCAM‐1 in human carotid plaques was detected by immunohistochemistry (Fig. 1A), western blotting (Fig. 1B) and immunofluorescence (Fig. 1C). The results indicated that the level of CKLF1 in human carotid plaques was higher compared to that in control group (*P* < 0.001). For VCAM‐1, the results showed that expression was also significantly higher than that in control group (*P* < 0.05).

### CKLF1 exaggerates neointimal hyperplasia in rats

**Fig. 2:**
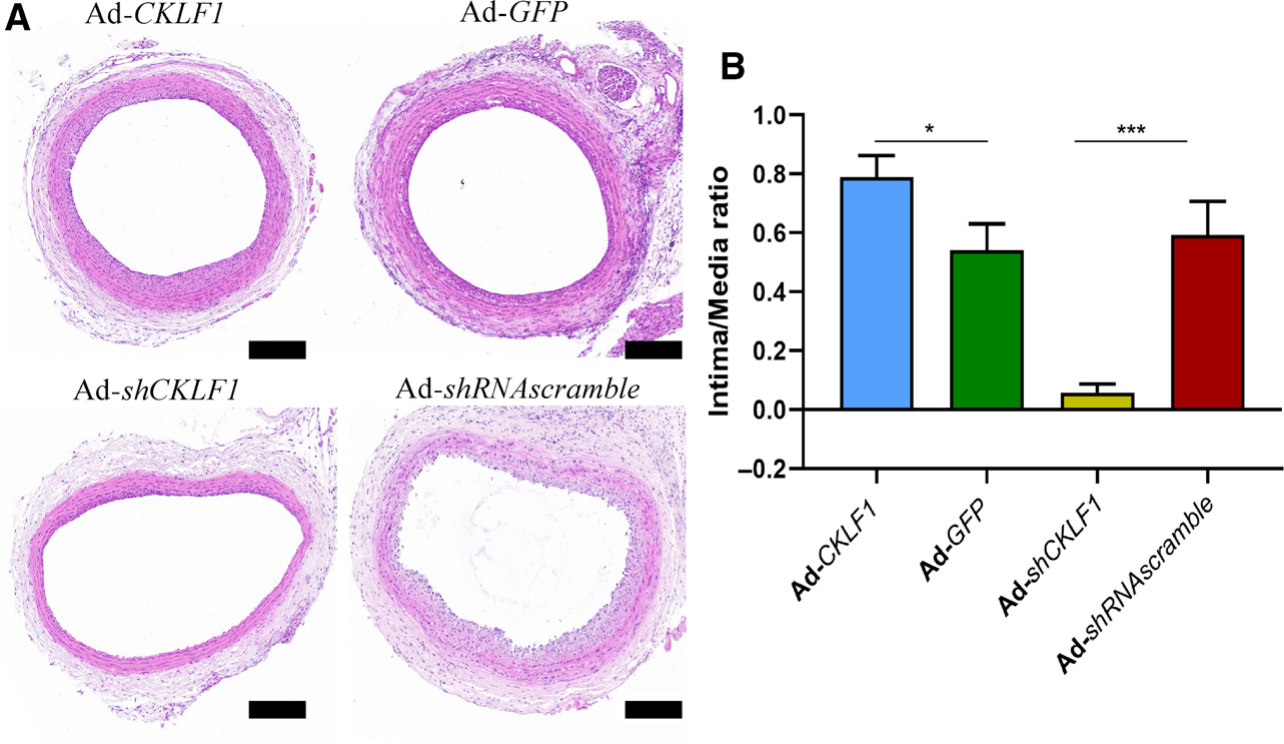
CKLF1 aggravates NIH after balloon injury. (A) The histological analysis of adenovirus‐transfected carotid artery was performed with HE staining. Scale bar = 200 μm. (B) Intimal/media ration in different groups. Adenovirus was injected into injured arteries and kept *in situ* for 30 min. The artery sample was harvested at 2 weeks after balloon injury. Data are shown as the mean ± SEM. Differences between two groups were analyzed by Student's *t*‐test (*n* = 5 independent experiments, *P < 0.05, ***P < 0.001).

Our previous study showed an upregulation of CKLF1 in NIH of rats [10]. Here, we aimed to explore the effects of CKLF1 overexpression on NIH *in vivo*. The HE staining showed that the NIH of rats in Ad‐*CKLF1* group was significantly higher than that in the control group (*P* < 0.05) (Fig. 2). To further confirm the role of CKLF1 in NIH, we designed Ad‐*shCKLF1* to observe whether CKLF1 knocking down could alleviate NIH. The results showed that knockdown of CKLF1 could effectively inhibit neointimal formation (*P* < 0.001) (Fig. 2). The intima/media ratio represents the severity of NIH of injured arteries.

### CKLF1 regulates the expression of VCAM‐1 in rat balloon‐injured arteries and HASMCs

**Fig. 3:**
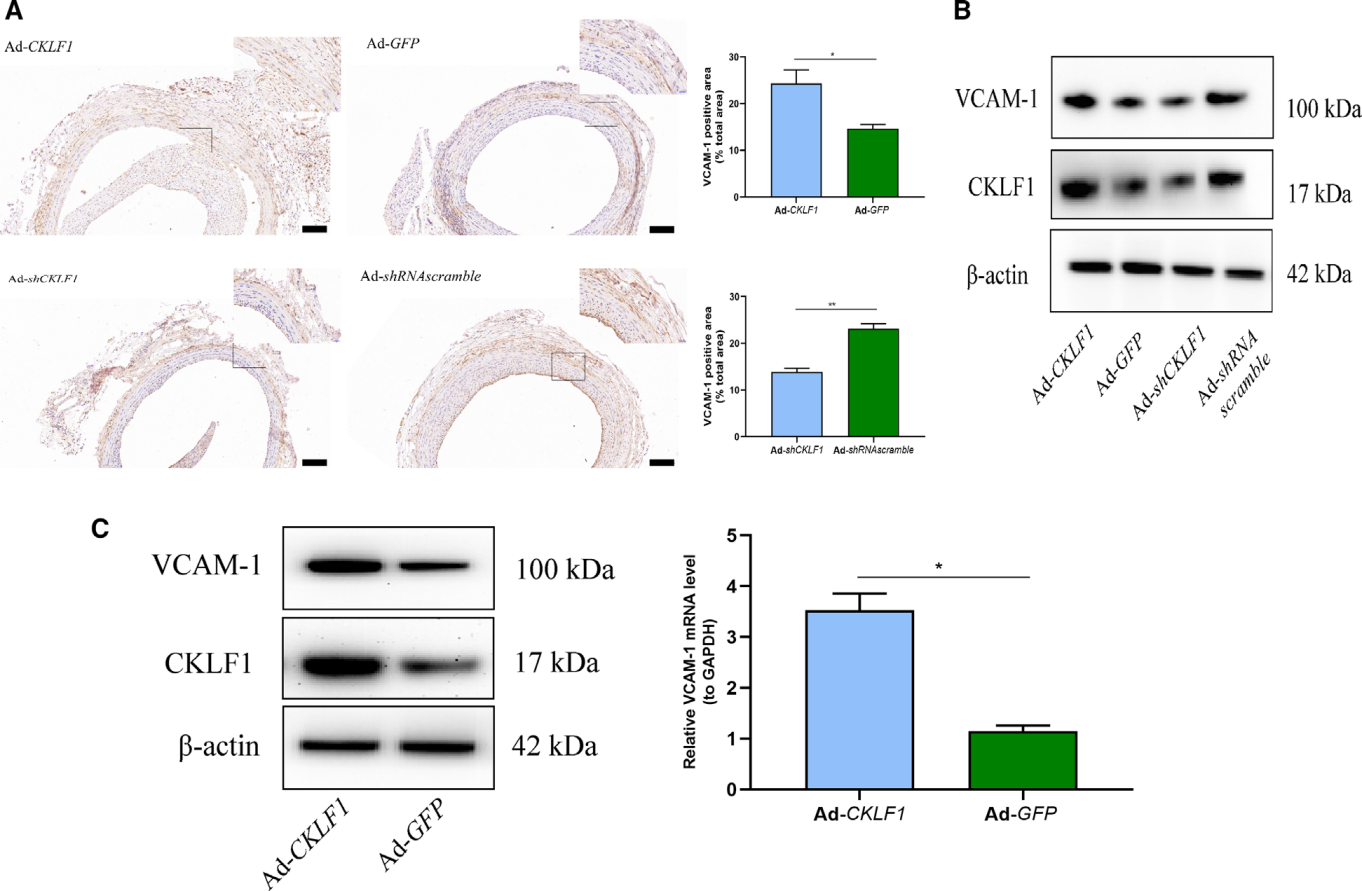
CKLF1 regulates the expression of VCAM‐1 in the rat carotid artery and HASMCs. (A) Immunohistochemical staining showed that CKLF1 overexpression upregulated the expression of VCAM‐1, whereas knocking down of CKLF1 downregulated the expression of VCAM‐1 in rats. Scale bar = 100 μm. Square areas are presented at 40× magnification. Data are shown as the mean ± SEM. Differences between two groups were analyzed by Student's *t*‐test (*n* = 5 independent experiments, *P < 0.05, **P < 0.01). (B) Western blotting analysis indicated that CKLF1 overexpression upregulated the expression of VCAM‐1, whereas knockdown of CKLF1 downregulated the expression of VCAM‐1 in rats (*n* = 3 independent experiments). (C) The mRNA and protein level of VCAM‐1 increased after Ad‐*CKLF1* transfection in HASMCs. For immunohistochemical staining, density values of positive areas were calculated for the statistical analysis. Data are shown as the mean ± SEM. Differences between two groups were analyzed by Student's *t*‐test (*n* = 3 independent experiments, *P < 0.05).

**Fig. 4:**
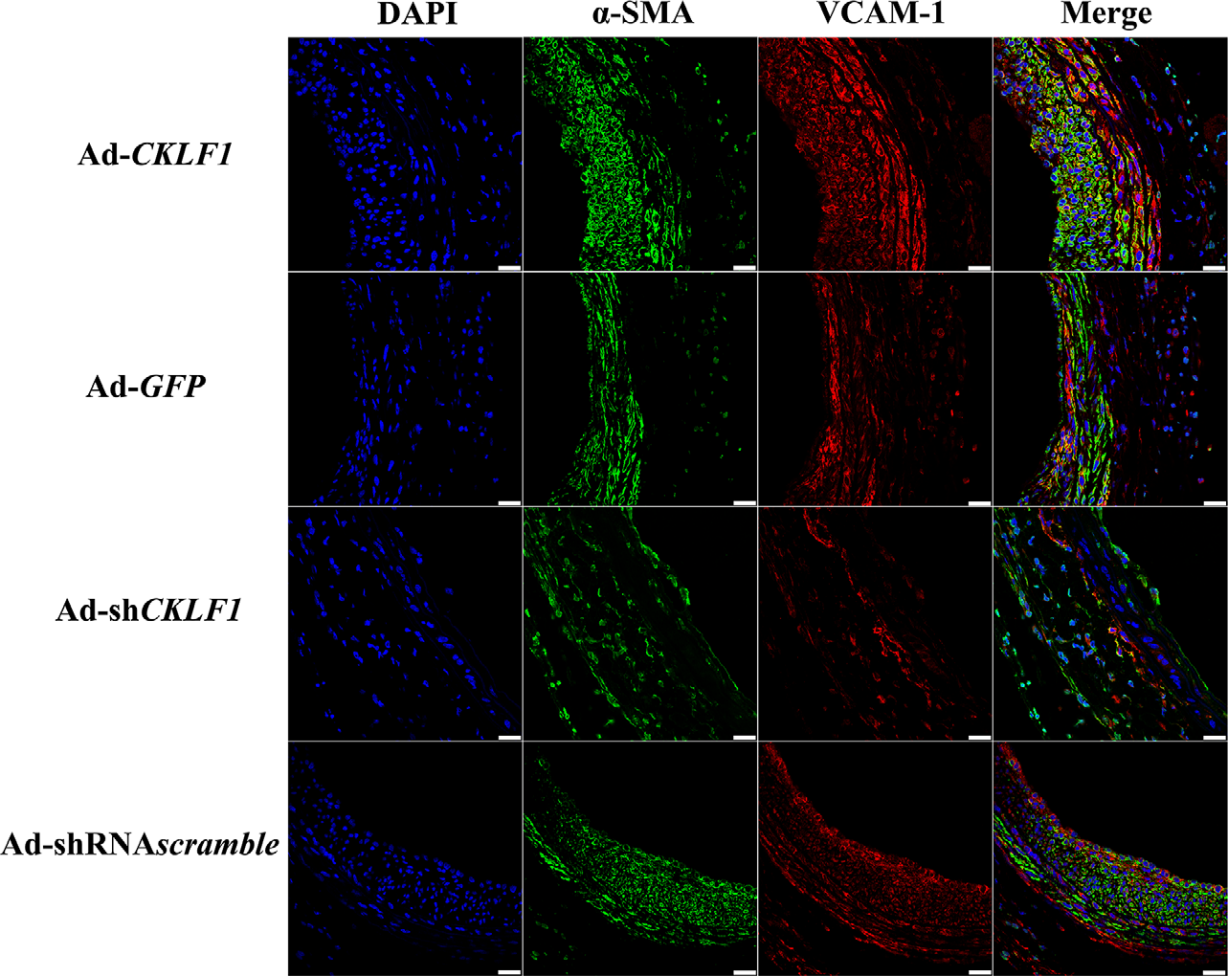
Immunofluorescence staining of rat carotid artery. Upregulation of CKLF1 is tightly associated with VSMC accumulation and thereby NIH in the rat carotid artery after balloon injury. Histological analysis of adenovirus‐transfected carotid arteries was carried out using anti‐SMA and anti‐VCAM‐1 antibodies and cell nuclei were stained with DAPI. The arteries were harvested 2 weeks after balloon injury (*n* = 5 independent experiments). Representative photomicrographs are shown. Scale bar = 25 μm.

Given the VCAM‐1 expression obviously increased in human carotid plaques, we aimed to confirm whether the CKLF1 could regulate the expression of VCAM‐1 in HASMCs and rats. The results obtained *in vitro* showed that CKLF1 overexpression could induce upregulation of VCAM‐1 at mRNA and protein levels (*P* < 0.05) (Fig. 3C). After that, we performed the *in vivo* experiments to explore regulation of CKLF1 on VCAM‐1 levels in rats. The results showed that CKLF1 overexpression could induce an upregulation of VCAM‐1 in rat balloon‐injured arteries compared to the control. Furthermore, the expression of VCAM‐1 was downregulated after CKLF1 knockdown (Fig. 3A,B and 4).

### CKLF1 upregulates VCAM‐1 through activating the NF‐κB pathway in HASMCs

**Fig. 5:**
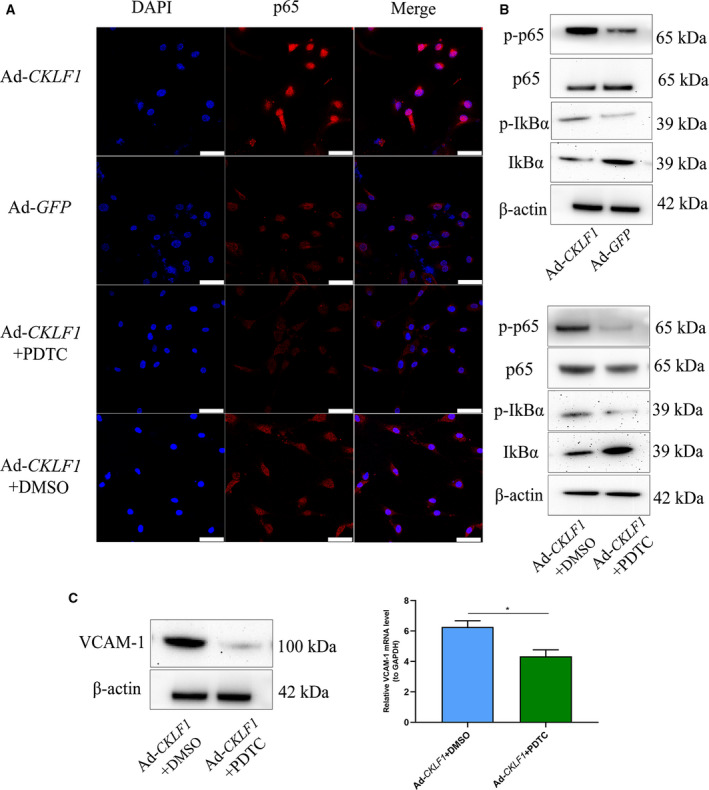
CKLF1 activates NF‐κB transcription factor and regulates the expression of VCAM‐1 via the NF‐κB signaling pathway in HASMCs. (A) Immunofluorescence results showed that CKLF1 overexpression promoted p65 nuclear translocation. The effect of nuclear translocation induced by CKLF1 was effectively inhibited by PDTC (50 μm). Cell nuclei were stained with DAPI. Scale bar = 50 μm (*n* = 3 independent experiments). (B) Western blotting analysis indicated that CKLF1 overexpression could induce an increase in the p‐p65 and p‐IκBa. By contrast, p‐p65 and p‐IκBa were inhibited by PDTC (*n* = 3 independent experiments). (C) PDTC intervention inhibited the upregulation of VCAM‐1 induced by CKLF1 at the mRNA and protein levels. Data are shown as the mean ± SEM. Differences between two groups were analyzed by Student's *t*‐test (*n* = 3 independent experiments, *P < 0.05). PDTC (50 μm). Dimethylsulfoxide (final concentration 0.1%).

Because CKLF1 could regulate the expression of VCAM‐1 *in vivo*, we explored their molecular mechanism. Given that NF‐κB is a ubiquitous transcription factor, VCAM‐1 is downstream of NF‐κB activation. Therefore, we performed a relative detection. As expected, the western blotting results indicated that overexpression of CKLF1 induced an increase in p‐p65 and p‐IκBa, which was abolished by PDTC (Fig. 5B). To further evaluate the effect of CKLF1 on the transcriptional activity of NF‐κB, we detected the nuclear transition of p65. The results showed that p65 was accumulated in nuclei after Ad‐*CKLF1* intervention compared to the Ad‐*GFP* group (Fig. 5A). To further confirm the regulation effect of NF‐κB on VCAM‐1, we investigated whether NF‐κB inhibitor, PDTC, could inhibit the increase in VCAM‐1 induced by CKLF1. As expected, p‐p65 and p‐IκBa were inhibited by PDTC (Fig. 5B) and VCAM‐1 overexpression was also inhibited (*P* < 0.05) (Fig. 5C).

### VCAM‐1 induced by CKLF1 promotes monocyte adhesion and migration of HASMCs

**Fig. 6:**
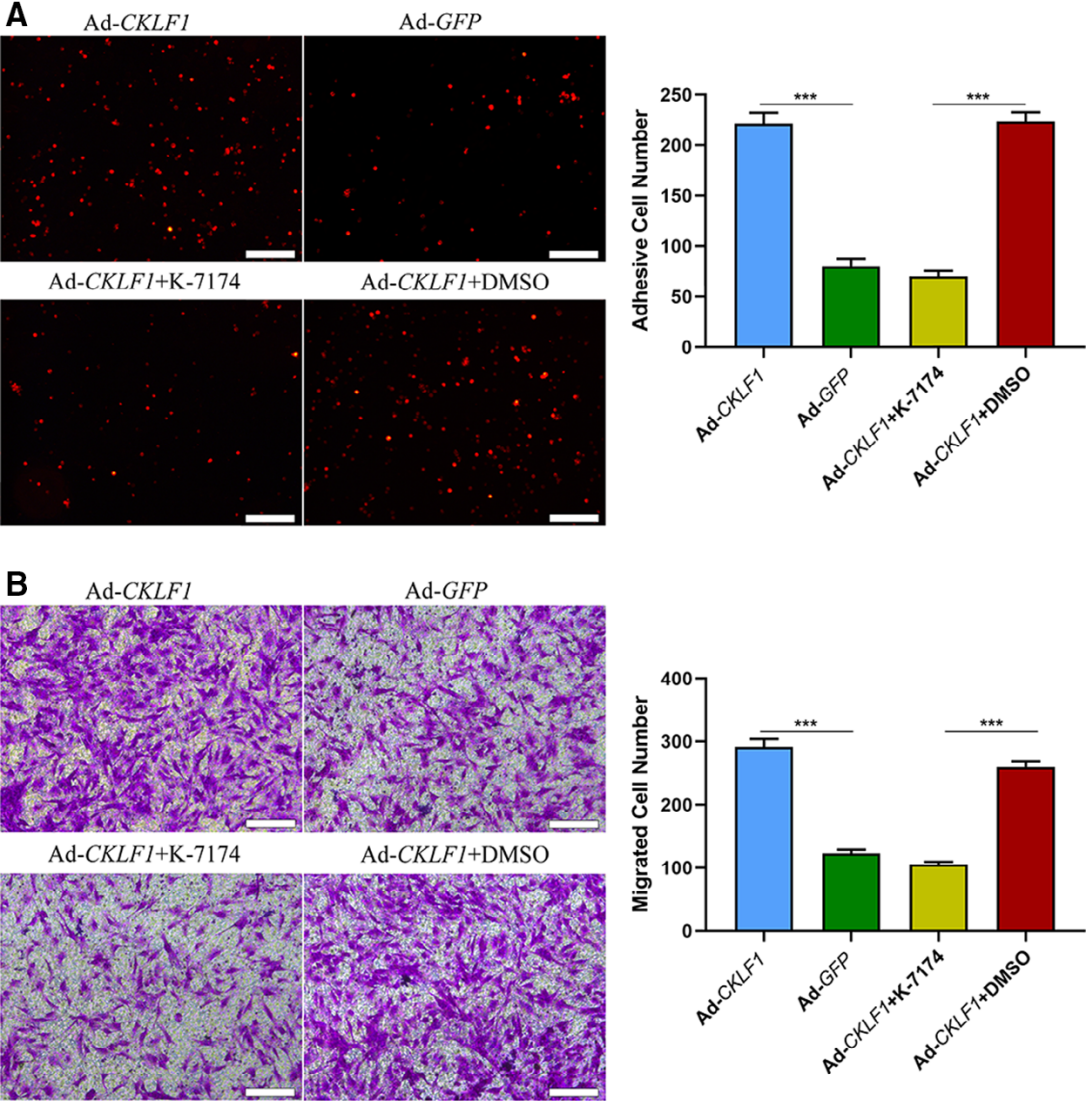
CKLF1 promotes monocyte adhesion and HASMC migration via VCAM‐1. (A) CKLF1 promotes U937 cell adhesion to HASMCs, and this adhesion effects can be inhibited by VCAM‐1 inhibitor (K‐7174). Scale bar = 200 μm. (B) CKLF1 could promote HASMC migration and HASMC migration can be inhibited by VCAM‐1 inhibitor (K‐7174). Scale bar = 200 μm. The migration assay was performed in Transwell inserts with a pore of 8 μm. Five areas were randomly selected and the cell numbers of adhesion or migration in each area were calculated for the statistical analysis. Data are shown as the mean ± SEM. Differences between two groups were analyzed by Student's *t*‐test (*n* = 3 independent experiments, ***P < 0.001). K‐7174 represents VCAM‐1 inhibitor (10 μm). Dimethylsulfoxide (final concentration 0.1%).

Given that CKLF1 could induce an increase in expression of VCAM‐1, we explored the role of VCAM‐1 in the process of monocyte adhesion and SMC migration. A monocyte adhesion assay showed that CKLF1 could significantly induce U937 cell adhesion to HASMCs compared to the control, and K‐7174 could effectively suppress U937 cell adhesion (*P* < 0.001) (Fig. 6A). CKLF1 showed the same effect on migration of SMC, and K‐7174 markedly inhibited SMC migration, as expected (*P* < 0.001) (Fig. 6B).

## Discussion

Consistent with our previous study, the expression of CKLF1 in human carotid plaques was upregulated compared to the control [10]. Interestingly, the distribution of CKLF1 and VCAM‐1 in plaques was parallel and both of them mainly focused around the cell‐enrichment areas. Recently, Kong *et al*. [19] reported that CKLF1 could induce an increasing of VCAM‐1 expression and accelerate ischemia/reperfusion injury. Therefore, we speculated that upregulated VCAM‐1 might be a possible mechanism to induce NIH via CKLF1. Given that SMCs were the most important cell type in hyperplastic intima, we first confirmed *in vitro* that CKLF1 could regulate the expression of VCAM‐1 in HASMC cell lines. The results indicated that CKLF1 overexpression can induce an increase in expression of VCAM‐1 at both protein and mRNA levels.

Accumulating evidence indicates that VCAM‐1 is involved in the process of atherosclerosis, as well as NIH [20]. In the present study, we found that the levels of VCAM‐1 in human carotid plaques increased obviously compared to the control. At the same time, we obtained similar results and trends for VCAM‐1 via immunofluorescence and western blotting analysis. By contrast, the results reported by Weinkauf *et al*. [21] indicated the VCAM‐1 was mainly expressed in the endothelial area of the carotid plaques, whereas our results indicated that the expression of VCAM‐1 was mainly located in the inflammatory cell areas. After analysis, their plaques were all from the symptomatic patients, which may explain the difference of VCAM‐1 distribution. Importantly, a previous study reported that VCAM‐1 was distributed in the endothelium of vessels in the early stage after injury, whereas its expression was mainly located in the medium of plaques at the late phase [22].

Next, we performed further experiments *in vivo* to confirm the impact of CKLF1 on VCAM‐1 expression. According to a previous study, balloon‐injured models were the most commonly used animal models for investigating NIH [23]. Thus, we used balloon‐injured rats to observe the impact of CKLF1 on VCAM‐1 expression. We found that the level of VCAM‐1 in CKLF1 overexpression group was higher than that in the control group. Importantly, the VCAM‐1 upregulation level was proportional to the severity of NIH after balloon injury. To further confirm the effect of CKLF1 on VCAM‐1 expression, we also knocked down the expression of CKLF1 in injured rat arteries, and the results showed that CKLF1 knockdown could downregulate the expression of VCAM‐1 and alleviate NIH after injury. Furthermore, a lack of endothelium was found in several carotid arteries after CKLF1 knockdown, which might be associated with CKLF1 knockdown promoted endothelial cell apoptosis [24]. However, the role of CKLF1 on the endothelium remains unknown.

To uncover the mechanism for CKLF1 in regulating the expression of VCAM‐1, we also perform the experiments using HASMCs cell lines. Our previous study had shown that CKLF1 could activate the NF‐κB transcription factor via PI3K/AKT signaling [11]. Given that VCAM‐1 is an NF‐κB dependent gene, we aimed to confirm that CKLF1 can impact the expression of VCAM‐1 via NF‐κB signaling. As expected, western blotting analysis indicated that CKLF1 overexpression induced an increase in p‐p65 and p‐IκBa, as well as in the expression of VCAM‐1. More importantly, we found that inhibition of NF‐κB activity induced a decrease in VCAM‐1 at the protein level, as well as at the mRNA level. However, the expression of VCAM‐1 in the CKLF1 knockdown group was not downregulated (data not shown). This might be associated with the low expression of CKLF1 in normal cells and other compensatory mechanisms of NF‐κB activation in the absence of CKLF1 [25]. In addition, immunofluorescence detection was performed to observe the effect of CKLF1 on the nuclear translocation of NF‐κB. The results obtained suggested that CKLF1 overexpression could induce p65 accumulation in nuclei and the NF‐κB inhibitor is able to effectively inhibit the translocation of p65. These results suggested that CKLF1 could regulate the expression of VCAM‐1 via NF‐κB signaling.

Recently, VCAM‐1 has been demonstrated to promote cell adhesion and migration of cancer metastasis [26]. Monocyte adhesion and migration of SMCs play a key role in the pathophysiology of NIH [27, 28, 29]. To date, it was unclear whether VCAM‐1 was involved in the process of NIH induced by CKLF1. In present study, we found for the first time that CKLF1 can increase U937 cell adhesion to SMCs. Importantly, U937 cell adhesion caused by CKLF1 could be partly inhibited by VCAM‐1 inhibitor (K‐7174). This suggested that CKLF1 can promote monocyte adhesion via VCAM‐1 and its mechanism of action may be by binding to the VLA‐4 receptor expressed on SMCs [30]. In addition, the results of the present study showed that CKLF1 could induce an increase in SMC migration, and K‐7174 can markedly suppress the SMC migration induced by CKLF1. Obviously, VCAM‐1 participates in the process of SMC migration induced by CKLF1, although its precise mechanism needs to be explored in the future. Indeed, we also investigated the effect of VCAM‐1 on CKLF1‐mediated proliferation of SMCs, although no obvious effect has been found (data not shown).

In conclusion, we have shown that CKLF1 can accelerate NIH by promoting monocyte adhesion and SMC migration partly through activation of the NF‐κB/VCAM‐1 pathway. These findings contribute to uncovering a novel mechanism by which CKLF1 accelerates NIH. However, there are some limitations to our study. First, only male patients or animals were included. The effects of sex‐specific differences in CKLF1‐driven NIH cannot be confirmed. Second, given that SMCs are the major cell types in NIH, we explored the impact of CKLF1 on SMCs. Indeed, other cell types such as leukocytes can also be involved in the process of NIH [31], which should be the focus of future research.

## Conflict of interests

The authors declare that they have no conflicts of interest.

## Author contributions

XL and CQ performed the experiments, analyzed the data and prepared the manuscript. YZ, RZ and LT analyzed and interpreted the results. CS, XZ and JF designed the study and revised the manuscript. All authors were involved in the discussions and all commented on the manuscript.

## Data Availability

The datasets generated and analyzed during the current study are available from the corresponding author on reasonable request.
